# What Are Health-Related Users Tweeting? A Qualitative Content Analysis of Health-Related Users and Their Messages on Twitter

**DOI:** 10.2196/jmir.3765

**Published:** 2014-10-15

**Authors:** Joy L Lee, Matthew DeCamp, Mark Dredze, Margaret S Chisolm, Zackary D Berger

**Affiliations:** ^1^Johns Hopkins Bloomberg School of Public HealthDepartment of Health Policy & ManagementBaltimore, MDUnited States; ^2^Johns Hopkins Berman Institute of Bioethics & Division of General Internal MedicineBaltimore, MDUnited States; ^3^Johns Hopkins UniversityHuman Language Technology Center of ExcellenceBaltimore, MDUnited States; ^4^Johns Hopkins University School of MedicineDepartment of Psychiatry and Behavioral SciencesBaltimore, MDUnited States

**Keywords:** communication, consumer health informatics, health information technology, social media

## Abstract

**Background:**

Twitter is home to many health professionals who send messages about a variety of health-related topics. Amid concerns about physicians posting inappropriate content online, more in-depth knowledge about these messages is needed to understand health professionals’ behavior on Twitter.

**Objective:**

Our goal was to characterize the content of Twitter messages, specifically focusing on health professionals and their tweets relating to health.

**Methods:**

We performed an in-depth content analysis of 700 tweets. Qualitative content analysis was conducted on tweets by health users on Twitter. The primary objective was to describe the general type of content (ie, health-related versus non-health related) on Twitter authored by health professionals and further to describe health-related tweets on the basis of the type of statement made. Specific attention was given to whether a tweet was personal (as opposed to professional) or made a claim that users would expect to be supported by some level of medical evidence (ie, a “testable” claim). A secondary objective was to compare content types among different users, including patients, physicians, nurses, health care organizations, and others.

**Results:**

Health-related users are posting a wide range of content on Twitter. Among health-related tweets, 53.2% (184/346) contained a testable claim. Of health-related tweets by providers, 17.6% (61/346) were personal in nature; 61% (59/96) made testable statements. While organizations and businesses use Twitter to promote their services and products, patient advocates are using this tool to share their personal experiences with health.

**Conclusions:**

Twitter users in health-related fields tweet about both testable claims and personal experiences. Future work should assess the relationship between testable tweets and the actual level of evidence supporting them, including how Twitter users—especially patients—interpret the content of tweets posted by health providers.

## Introduction

**Figure 1:**
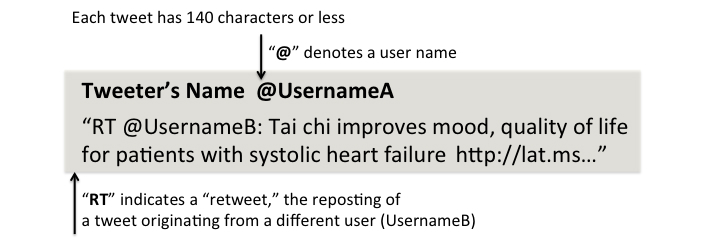
Anatomy of a tweet.

Close to 90% of US adults use the Internet [1]; 72% of those visit social media websites [2]. In fact, 12% of Internet users access social media to research health issues [3]. In particular, the micro-blogging service Twitter, which enables users to send short messages no more than 140 characters in length (see Figure 1 for the breakdown of a tweet), has quickly grown in popularity, with an estimated 255 million users monthly and 22% of active accounts based in the United States [4].

Health providers, patients, and patient advocates comment on a diverse array of topics on Twitter [5-7]. Previous reports have examined discrete health topics that users are discussing on Twitter [8-10], as well as concerns related to medical professionalism [11,12]. Health researchers have also used Twitter as a tool to track disease outbreak [13,14] and recruit study subjects [15]. However, little in-depth knowledge is available about health-related content by health professionals on Twitter, in particular whether such content represents personal opinion, a claim that those who view the tweet would expect to be supported by rigorous evidence, or something else. In addition, despite recent controversy over whether physicians should separate their “personal” and “professional” identities online [16-18], little is known regarding health professionals’ actual behavior.

Filling these knowledge gaps requires investigating message content at a depth only possible through qualitative analysis of health professionals’ tweets. This paper reports the results of a content analysis of such tweets. The primary objective of the study was to describe the general types of content on Twitter authored by health professionals, focusing on health-related content. Key content types of interest included content that would be perceived as “personal” and content that users would expect to be supported by medical evidence (ie, “testable” claims about health). Secondary objectives included comparisons of content areas by user type (eg, providers, health advocates). Based on prior experience and existing professional guidelines, it was hypothesized that self-identified health professionals would not post personal content on Twitter and that testable claims would be rare.

## Methods

### Data Collection

Data collection occurred in three basic steps. First, health users of Twitter were identified. Lists of Twitter users created by Organized Wisdom [19], a company in the health information technology sector, were used to gather data. Organized Wisdom collates health-related Twitter users into lists that enable others to more efficiently find information online; for example, the “doctors verified” list consists of physicians who openly disclose their credentials online. Of these lists, 37 are Twitter accounts for doctors, nurses, hospitals, and other health organizations that cover seven user groups of interest: health advocates, businesses, health care organizations, government organizations, students, professionals, and other (eg, health care-related publications).

Second, from these health users, an equal number (n=200) of the most recent publicly available tweets for each user in each group, including retweets, was downloaded during the period July 31-August 3, 2012. In cases where users had fewer than 200 tweets, all available data were obtained.

Third, from this large collection of tweets from the seven user groups, a smaller collection was sampled. For each group, a user from the group was randomly selected, and a random tweet (without replacement) was selected for that user. This strategy was used to create a preliminary sample of tweets (15 random tweets from each user group, or 105 total) to develop a coding scheme and a final sample for in-depth qualitative analysis (100 random tweets from each user group, or 700 total).

### Content Analysis

The research team first hypothesized content areas of interest from their own experience and the published literature to generate a draft coding scheme. Then, using the preliminary sample of 105 tweets, 4 coauthors (JL, ZB, MD, MC) conducted content analyses of each tweet using this draft scheme. The coding scheme was then revised for the final analysis. The categories of interest are presented in Table 1. Subsequent analysis was then conducted on an additional 700 tweets as described above. Areas of disagreement between reviewer pairs were resolved by consensus in a case-by-case fashion. Reviewers were blind to the identity of the Twitter users during content analysis.

**Table 1:** Categories of health-related tweets identified via content analysis (n=346; content frequency may be greater than 100% because content areas were not mutually exclusive).

Tweet type	Definition	Example	Frequency among health tweets, %
Testable	An evidence-based claim whose veracity could be assessed	RT @username: Did you know? You can become more sensitive to alcohol as you get older: http://[link] #aging	53
News	Report of information or events that happened recently, often refers to traditional news outlet	“@username: State charges man with #stealing $400,000 from church, #Alzheimer’s patient, choral group http://[link] #embezzle	41
Commercial	Refers to or advertises a service or product for sale	RT @username: Learn to capture/edit/produce quality videos at ASGE’s Video Editing Course for Physicians, June 8-10, Oak Brook, IL. ...	27
Wellness	Refers to food, diet, or exercise	RT @usernameReady for playoff football? Enjoy the wild card games & stick to a healthy diet with these recipes: http://[link]	14
Personal	Refers to personal experiences relayed by users	I visited @username today for the first time. Cool space but where are the women? Let’s get the women who made the @username pledge going!	18

After unmasking the data, users and user groups were reviewed to verify the accuracy of categories provided by Organized Wisdom. None of the tweets reviewed were found to be authored by the research team. In several instances, the provided categories were inaccurate; for example, a self-identified hotel was miscategorized as a government agency. Therefore, two members of the research team (JL and ZB) re-categorized all users into seven new user groups based on consensus review of publicly available profiles (Table 2): users without available profiles, non-health users, health advocates, health businesses, non-provider health professionals, health providers, and health care organizations. This resulted in a different number of tweets for each of the seven groups.

**Table 2:** Types of users.

User type definition	User examples	Users (n=255), % (n)	Health tweets (n=346), n	Tweet examples
No profile: Accounts deleted or suspended after data collection so profiles could not be assessed	Not available	7 (19)	19	Vision Therapy Improves Vision Related Learning Problems: LIVONIA, MICHIGAN -- When children are having trouble... http://[link]
Non-health related users: Users unaffiliated with a health profession, organization, or mission	Justin Bieber fan account; a hotel in Gloucestershire, England; expert in “online promotion”	25 (64)	58	RT @username: These Baked Honey-Mustard Chicken Bites r big on flavor - and small on POINTS® Value! http://[link]
Health advocates: Users educating and advocating health issues on behalf of patients	Patient advocate with diabetes; educator and promoter on disabilities issues	6 (16)	26	My Life as Mandy... with Epilepsy: Educational Information http://[link]
Non-provider health professionals: Users working in the health field who do not provide direct patient care	Health economist; registered dietician; medical student	11 (28)	43	Hey girl, I so hear ya! I'm on the same boat! RT @username: ABSOLUTELY NEEDS TO LOSE WEIGHT!! I CANT KEEP PROCRASTINATING! Get eht 2qethr.
Health providers: Direct-care providers, such as physicians and nurses	A registered ER nurse “and mother of 3”; a clinical cardiologist; “physician, keynote speaker, and media health expert”	22 (56)	59	Human factors:articulate video on patient safety inspired from personal loss http://[link]
Health businesses: Commercial businesses selling specific service or product	Herbal supplements company; health care marketing agency	22 (55)	99	@username Good luck with all that reading. Have you seen our Anatomy & Physiology Online? Makes learning easier http://[link]
Organization: Hospitals, medical societies, clinics, and journals	The Cancer Letter; Albert Einstein College of Medicine; Kids in Need of Dental Services	7 (17)	42	RT @username: Public #malaria drug research #data now more discoverable http://[link] #open #science20

### Statistical Analysis

Descriptive statistics were used to tabulate types of tweets for each group of users. Statistically significant differences between the proportion of tweets among user groups were assessed using the chi-square test. Stata 13 was used for the analysis.

This study was reviewed and declared exempt from further review by the Johns Hopkins Medicine Institutional Review Board.

## Results

### Tweets Overall

**Figure 2:**
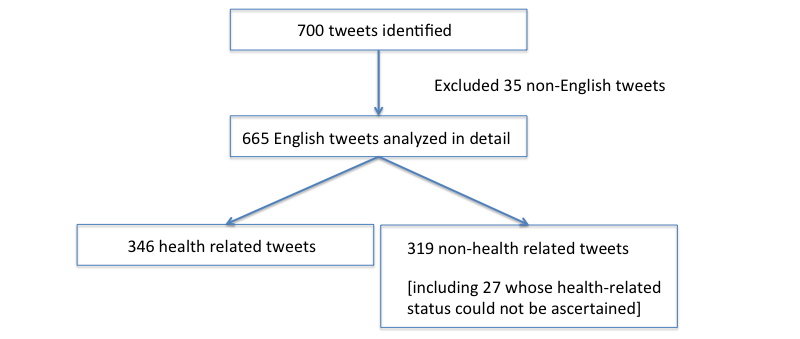
Inclusion and exclusion of tweets.

Of all tweets, 5.0% (35/700) non-English tweets were excluded. The remaining 95.0% (665/700) English-language tweets were analyzed in depth (Figure 2). A total of 255 unique users contributed to the 665 tweets. Of the tweets in the analytic sample, 52.0% (346/665) were categorized as health-related. For example, a tweet such as “RT @Username: Tai chi improves mood, quality of life for patients with systolic heart failure” was categorized as health-related. Each health-related tweet was thereafter sub-coded into at least one of six non-mutually exclusive categories, as defined by Table 1.

Nearly one-third (31.1%, 207/665) of all tweets in the sample were personal in nature. Among personal tweets, 70.5% (146/207) were non-health related, whereas 29.5% (61/207) were health-related. This finding was statistically significant (*P*<.001).

### Content Analysis of Health-Related Tweets

The research team was most interested in health-related tweets. Just over half (53.2%, 184/346) of these tweets contained “testable” claims, defined as claims for which someone viewing the tweet might expect to be supported by medical evidence. An example of a testable tweet is shown in Table 1 (first row).

Within health-related tweets, news was the second most frequent content category; 41.0% (142/346) of these tweets contained news. Over a quarter (26.9%, 93/346) contained reference to a commercial product or service, 17.6% (61/346) relayed a personal viewpoint or experience, and 17.1% (59/346) discussed wellness.

### Categories of Health-Related Tweets by User Type

#### Overview

The analysis also compared the types of health-related tweets made by each of the seven user groups. Many of the users generating non-English tweets (9/14) contributed English tweets as well. The characterization of the health-related tweets by each user group is as follows.

#### No Profile

For 7.5% (19/255) of users, contributing 5.1% (34/665) of tweets, the user profile could not be accessed. Of these tweets, 56% (19/34) had health-related content. Among health-related tweets, 68% (13/19) made testable claims. Because 32% (6/19) of tweets by this group were personal, some of these users may have been individuals.

#### Non–Health-Related User

During the analysis, it was noted by the research team that—although users in the health field were originally intentionally sampled using Organized Wisdom—about a quarter of the users in the analysis (62/255) were not affiliated with a health profession, organization, or mission. This was the largest user group in the sample and potentially represents a comparison group of non-health users. These users contributed 30.2% (201/665) of all tweets in the analysis and 16.8% (58/346) of health-related tweets.

Non-health users tweeted more about wellness than health-related users: 33% vs health user average of 14%, *P*<.001; health users defined as all groups other than non–health-related users, including no profile, health providers, health-related businesses, non-provider health professionals, patient advocates, and organizations. More so than any other group, the majority of tweets by this group also included a link (86%, 50/58), and significantly fewer of those links were accessible by the research team (43%, vs health user average of 80%, *P*<.001). Of all tweets by these users, 55% (32/58) had a testable claim and 47% (27/58) were news.

#### Health Providers

Direct-care providers, such as physicians and nurses, encompassed the next largest group of users, accounting for 23.1% (59/255) of the analytic sample. These users contributed 14.4% (96/665) of tweets analyzed; 61.5% (59/96) of tweets by these users were health-related in nature. They shared testable claims and news (61%, 36/59 and 56%, 33/59 respectively) more than other groups of individual users, although 14% (8/59) also mentioned a commercial product or service and 17% (10/59) were personal (eg, “That awkward moment when there is no awkward moment to tweet about”).

#### Health-Related Businesses

About one-fifth of the users (21.6%, 55/255) in the analysis were health-related businesses whose health-related tweets constituted 28.6% (99/346) of the analytic sample. Many tweets by health businesses (44%, 44/99) mentioned a product or service, the highest percentage among the categories of users. Aside from these commercially oriented tweets, businesses often shared information seemingly relevant to their market (eg, “When You Leave Someone with a Mental Illness” from an addiction center or “#Pharma cos. push for social-media rules #hcsm #hcmktg #web20” from a marketing agency). These users also had significantly more tweets that were personal in content (8% vs health user average of 19%, *P*=.003) than other health users.

#### Non-Provider Health Professionals

Non-provider health professionals are those in health care professions who do not provide direct patient care. Non-provider professionals made up 11.0% (28/255) of users in the sample, including two medical students; 12.4% (43/346) of total health tweets came from this group. The two medical students contributed nearly half of the tweets in this group (47%, 20/43). Only 49% (43/87) of tweets by this group of users were health-related, which was the lowest percentage among health users. This group of users made the fewest testable claims and referenced the news the least in their health tweets as compared to all other user groups. Instead, nearly half of the content (44%, 19/43) was about the users’ personal experiences. Among the two medical students, 70% (14/20) of health tweets were personal in nature. At the same time, this group of users also tweeted less about commercial products or services compared to any other group.

#### Patient Advocates

Patient advocates made up 6.3% (16/255) of the users in our analysis, and together they contributed 6% (45/665) of tweets. More than half (58%, 26/45) of tweets by these users were health-related; 38% (10/26) of the users’ health-related tweets were personal in nature, the second-highest number of personal tweets among the groups. Only 8% (2/26) of health tweets by this group touched upon wellness, which was below the average of 14%. Many of the messages retweeted content from other users, and 85% (22/26) included a link.

#### Organizations

Health care organizations represented in this sample were hospitals, medical societies, health care clinics, and journals. These users represented 5% of our sample and contributed 7.7% (51/665) of tweets. More than half (82%, 42/51) of their tweets were health-related. Moreover, these health tweets were most likely to share “testable” claims than other users. The majority of organizations’ tweets contained testable claims (64%, 27/42) and news (52%, 22/42), and few were personal in nature (7%, 3/42). Yet organizations also had a high number of commercial tweets; a third of their health tweets (33%, 14/42) advertised a commercial product, or most often, their own services (eg, CPR courses, lab tests, and staff education curriculum).

Like tweets by patient advocates, tweets by health care organizations often passed on information through links. Most of their links (91%, 32/35) were accessible, compared to an overall average of 72% (*P*=.04).

### Group Comparisons

Table 3 summarizes the distribution of tweet content areas among user groups, where content by health providers serves as the baseline for comparison. Overall, there were no significant differences in the types of content between health providers and non-health users and those without available profiles.

Health advocates tweeted about personal health matters significantly more than providers. Provider tweets were most different from those of health care businesses. Businesses were statistically more likely to tweet about news and less likely to tweet about wellness, and non-provider health professionals were statistically more likely to tweet testable claims and news stories and less about personal content. The tweets of health care organizations and providers differed as well. Organizations’ tweets were statistically more likely to include commercial content and wellness but were less likely to include personal content.

**Table 3:** Percentage of health-related tweets in selected content areas by user group.

	Testable	News	Commercial	Wellness	Personal
Providers	61	56	14	25	17
No profile	68	32	16	10	32
Not health	55	47	22	33	9
Business	47	34^a^	44^a^	12^a^	8
Non-Providers	37^a^	26^a^	16	12	44^a^
Advocate	50	35	15	7	38^a^
Organization	64	52	33^a^	10^a^	7^a^

^a^Statistically significant difference compared to the provider group at *P*<.05 using individual chi-square comparisons.

## Discussion

### Principal Results

This analysis of self-identified health users and their health-related messages on Twitter identified several important findings. First, over half of the tweets in the sample included tweets with testable claims, defined for this study as content that a Twitter user might expect to be supported by medical evidence. The majority of health tweets were testable and comprised various topics. In a preliminary analysis, we found that about 40% of such testable tweets shared medical advice while 23% disseminated scientific news or research books and articles. Although the research team did not analyze this in detail, the sources behind these medical claims were more varied than scientific research (ie, “evidence-based” medicine claims), ranging from common sense (eg, advice to drink more water on a hot day) to WebMD to Dr. Oz to personal blogs. Whether the claims were actually scientifically valid was not examined and requires further study.

Second, in addition to traditional providers, a wide range of non-health providers are sending health-related tweets. Moreover, the content of tweets by health-related users on Twitter varied with user type. Health care organizations, for example, shared more health news and testable claims than individuals but also endorsed commercial services or products more than individuals. In the future, it could be important to examine whether organizations’ tweets are based more often in rigorous evidence as compared to those of individual health professionals. These findings confirm and expand upon the work of Sugawara et al [20] and Chretien et al [21], who both focused on a subset of health users. In examining the tweets of cancer patients in Japan, Sugawara found that patients tweeted about both personal (eg, greetings, chats) and medical matters (eg, information about treatments). Similarly, Chretien found that health providers (specifically, physicians) shared personal, commercial, as well as medical information over Twitter.

We found that 17% of users in this sample were non-provider individuals in the health field, together contributing 20% of health-related tweets. Their participation illustrates the wide range of non-providers engaging in information sharing and health messaging on Twitter. While this community of users represents a significant resource of health conversation and information on Twitter, these non-providers are not bound by professional guidelines on Twitter use. It is unclear how Twitter users might use or understand the tweets of this group compared, for example, to tweets by physicians and nurses. Given that trust is a significant feature of social networks (with young users potentially trusting their social network as much as physicians), this requires further examination [3,22].

Third, these findings demonstrate potential gaps between professional guidelines and online behavior [23,24]. Many medical societies, institutions, and medical schools disseminate such guidelines for clinicians and students. Often, these guidelines encourage the use of social media as a tool for endorsing medical information, discourage using the medium for medical consults, and counsel against posting personal content. Despite this, 17% of health tweets from providers and 74% from medical students included personal content. This lack of adherence to professional guidelines identifies an area where additional education or potentially consensus building within the profession is needed. Similarly, the sizable minority (14%) of providers’ tweets that included commercial products or services confirms prior findings and concerns about potential conflicts of interest on Twitter (a medium where disclosure is difficult, if not impossible) [21,25].

As the use of Twitter has increased, so has the scholarship on the use of Twitter for health-related purposes. Some researchers have used Twitter as a surveillance [7,13,26] and prediction tool for disease outbreak [27] while others have characterized the relationship between segments of health-related users on Twitter, such as medical societies [28], internal medicine physicians [29], and cancer patients [20]. Though few papers examined health-related users as a whole, or the content of their tweets in detail, ours is not the first content analysis of Twitter. For example, Chew and Eysenbach analyzed individual tweets relating to the 2009 H1N1 pandemic, finding that information from a variety of credible sources, in addition to opinions and experiences, were disseminated on Twitter [26]. Our study, a qualitative analysis of tweets from health-related users, addressed different questions and thus made use of a different approach.

This study adds to the growing literature on using Twitter for health purposes by examining the content of tweets by health-related users. In doing so, this study had several important strengths. It represents an in-depth exploration of the types of health-related users and content on Twitter. The analysis is the first to examine in detail both health users and their health-related content on Twitter, orienting researchers and clinicians to the Twitter health landscape. While previous studies focused on Twitter use among certain populations or users [8,20,21,30,31] such as cancer patients in Japan, or certain topics such as childhood obesity [9], this study explored several types of users, achieving breadth and qualitative depth simultaneously.

### Limitations

The study was not without limitations. Data collection occurred in the summer of 2012; this sample thus reflects a Twitter landscape that may have shifted since then. For example, adherence to professional guidelines could be expected to increase over time. This study’s findings set a baseline for future analyses. The selection of the analytic sample was limited by the absence of a simple mechanism to sample health-related users and tweets on Twitter. For this reason, it was not feasible to select a random sample of all health users and their tweets, reducing the generalizability of the study findings. Although Organized Wisdom, by organizing content into lists, seemed to offer a reasonable way to address this problem, many user category designations were inaccurate. Favoring accuracy of user type over preservation of the original categories, the research team undertook its own characterizations. Because this reduced the number of tweets in certain user groups, this reduced the ability to determine statistical significance between groups. If so, the results likely underestimate potential differences between groups. Importantly, this only reinforces the need for in-depth analysis while revealing a concern that collated “health-related user” content might not be from health-related users.

The analysis did not purposely evaluate “high impact” users (eg, Dr. Oz, Atul Gawande, Sanjay Gupta). We also did not independently verify user profiles, and users representing themselves as providers were not checked against other databases. Furthermore, while the analysis was double coded and disagreements were resolved by consensus, the subjective nature of coding limits the validity of these findings.

### Future Considerations

This study presents several opportunities for further research. While a preliminary assessment evaluated the evidence base of each testable tweet, future analysis could examine this subset of tweets (health-related tweets that contain testable claims), for example, by comparing them to accepted strength of evidence standards [32]. Cross-referencing the identities of the users would be another next step in refining the characterization of users, while including Twitter users who are verifiable, self-identified patients or providers would further enrich the analysis of health messages.

### Conclusions

In conclusion, health-related users and the content they share on Twitter are diverse. While providers’ tweets often include testable claims, they also make use of personal statements. Non-providers also contribute to ongoing health-related messaging on Twitter. Users in health-related fields who are actively engaging in health-related conversations on Twitter, as well as those who are merely reading these users’ tweets, should be oriented to the diversity of health-related Twitter content, and, if possible, to the validity of associated tweets.
